# An ER–microtubule bridge: Reticulon 17 links microtubules with ER network organisation in plants

**DOI:** 10.1111/tpj.71028

**Published:** 2026-07-04

**Authors:** Carmen Mata, Stefan Wojcik, Verena Kriechbaumer, Charlotte Pain

**Affiliations:** ^1^ Endomembrane Structure and Function Research Group, School of Biological and Medical Sciences Oxford Brookes University Gipsy Lane Oxford OX3 0BP UK; ^2^ Oxford Brookes Centre for Bioimaging Oxford Brookes University Gipsy Lane Oxford OX3 0BP UK

**Keywords:** endoplasmic reticulum, Reticulon 17, microtubule cytoskeleton, ER structure, RHD3, confocal microscopy

## Abstract

The plant endoplasmic reticulum (ER) forms a highly dynamic tubular network whose architecture depends on ER‐shaping proteins and its interaction with the cytoskeleton. While actin is well known to drive ER movement in plants, how the ER associates with microtubules and how this affects ER network architecture remain poorly understood. Here, we identify *Arabidopsis thaliana* reticulon 17 (RTN17) as an atypical reticulon that links the ER to the microtubule cytoskeleton. RTN17 features extended, intrinsically disordered N‐ and C‐terminal domains enriched in low‐complexity regions, consistent with a scaffolding or hub function. Topology analysis using redox‐sensitive roGFP2 constructs shows that both termini face the cytosol, yet RTN17 lacks the amphipathic helix typical of ER‐shaping reticulons and does not induce membrane constriction. Instead, RTN17 localises to punctate foci on curved ER membranes, recruits the ER fusogen ROOT HAIR DEFECTIVE3 (RHD3), and co‐expression alters ER architecture and dynamics. RTN17 puncta preferentially co‐localise with microtubules, and its over‐expression promotes ER alignment with the microtubule network.

We propose that RTN17 acts as a multifunctional scaffold linking curved ER domains with the microtubule cytoskeleton and localising RHD3 to these sites to regulate ER fusion events. By integrating curvature sensing, cytoskeletal attachment and fusion regulation, RTN17 represents a new class of plant reticulons with scaffolding rather than shaping functions. This work highlights an unrecognised mechanism coordinating ER organisation with the cytoskeleton, providing insights into how plants achieve spatial control of endomembrane architecture and potentially adapt membrane dynamics to developmental or stress cues.

## Significance Statement

Endoplasmic reticulum (ER) organisation underpins key processes in plant growth, secretion, and stress responses, yet how the ER connects to the cytoskeleton remains unclear. We identify Arabidopsis Reticulon 17 (RTN17) as an atypical reticulon that links the ER to microtubules and recruits the ER fusogen RHD3 to curved ER domains. RTN17 reveals a previously unknown mechanism that coordinates membrane architecture, cytoskeletal alignment and fusion activity in plant cells.

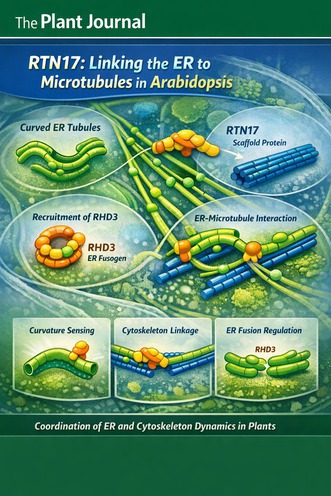

## INTRODUCTION

The plant endoplasmic reticulum (ER) is a highly dynamic membrane‐bound organelle that forms a complex network throughout the cytoplasm (Ridge et al., 1999). The ER can be divided into two structurally distinct subdomains: tubules that interconnect at three‐way junctions and cisternae which appear to be expanded flattened regions of the ER. This structure encloses polygonal regions of cytoplasm, which themselves are a reflection of the structure of the ER. The proportion of these different structural subdomains is altered in response to numerous conditions, including the biosynthetic requirements of the cell (Stephenson & Hawes, 1986), pathogen attack (Hardham et al., 2008), and both biotic and abiotic stresses such as heat shock (Pain et al., 2019).

The ER interacts with both the actin and microtubule cytoskeleton. The actin cytoskeleton and an associated class of XI‐K myosins are known to contribute significantly to the movement of the plant ER (Sparkes et al., 2009). Over‐expression of loss‐of‐function mutants of class XI‐myosins abolishes ER streaming and gross movement (Griffing et al., 2014; Ueda et al., 2010). The role of the microtubule cytoskeleton in ER dynamics is much less well understood though it has been implicated in slow ER tubule extension (Hamada et al., 2014). It has also been suggested that the microtubule cytoskeleton may support branch points in the ER, contributing to three‐way junction formation (Hamada et al., 2014). This is a much more minor contribution to ER dynamics than has previously been reported in mammalian cells, where a significant proportion of ER dynamics have been attributed to interactions with the microtubule cytoskeleton (Friedman et al., 2010; Kors & Schlaitz, 2024; Lee et al., 1989; Lee & Chen, 1988; Terasaki et al., 1986; Waterman‐Storer et al., 1995).

The structure of the plant ER is controlled by three classes of ER shaping proteins: the Lunapark (LNP) protein family (Kriechbaumer, Breeze, et al., 2018; Ueda et al., 2018), ROOT HAIR DEFECTIVE3 (RHD3) (Chen et al., 2011; Zhang, Wu, et al., 2013) and the reticulon (RTN) protein family (Sparkes et al., 2010; Tolley et al., 2010). The LNP family plays a role in controlling the size of ER cisternae and the relative proportion of cisternae in the ER (Kriechbaumer, Breeze, et al., 2018), whilst RHD3 is a putative ER fusogen, contributing to the formation of three‐way junctions and ER connectivity (Chen et al., 2011, Zhang, Wu, et al., 2013). RHD3 is a plant homologue of mammalian atlastin (ATL) and yeast Sey1p (Chen et al., 2011), which have been shown to mediate *in vivo* homotypic ER fusion in a GTP‐dependent manner (Anwar et al., 2012) as well as being sufficient to produce a reconstituted network via tubular fusion *in vitro* (Powers et al., 2017).

The Arabidopsis RTN protein family is one of the largest ER shaping protein families, comprised of 21 members, with the majority of RTNs involved in ER tubule maintenance and ER membrane constriction (Sparkes et al., 2010). However, the Arabidopsis RTN proteins have been implicated in a wide range of functions, including lipid regulation (Kriechbaumer, Breeze, et al., 2018), roles at ER‐plasma membrane contact sites and plasmodesmata (Knox et al., 2015; Kriechbaumer, Botchway, et al., 2015; Wang et al., 2023), and autophagy (Zhang et al., 2020). In mammalian cells, RTN proteins have also been linked to exocytosis (Mukherjee & Levy, 2019), nanodomain organisation on ER tubules (Gao et al., 2019), and control of axon regeneration in the central nervous system (Diekmann et al., 2005).

Arabidopsis RTN protein family members are identified by the presence of a conserved RTN homology domain (RHD), which is approximately 170‐190aa in length (Nziengui et al., 2007). The RHD Is composed of two hydrophobic regions that are linked by a short hydrophilic link (Hu et al., 2008; Tolley et al., 2010; Voeltz et al., 2006). Whilst the hydrophobic regions insert into the ER and form a classic ‘W’ shaped structure, the remainder of the protein is localised to the cytosol (Tolley et al., 2010). This ER shaping function of some RTNs is supported by the presence of an amphipathic helix (APH) which is located C‐terminally to the RHD, and works with the RHD to wedge the ER membrane, inducing ER membrane bending (Breeze et al., 2016; Brooks et al., 2021; Cui et al., 2011; Jensen et al., 2011). However, the molecular players connecting the ER to the microtubule cytoskeleton in plants remain largely uncharacterised.

In this study, we describe a novel function for the reticulon‐family member RTN17. Unlike canonical RTNs, RTN17 possesses extended N‐ and C‐terminal domains that equip it to act as a functional hub, linking the ER to the microtubule cytoskeleton. Rather than acting primarily as an ER‐shaper, we demonstrate that RTN17 engages the ER‐fusing GTPase RHD3 and cooperates with the underlying microtubule cytoskeleton to modulate ER architecture and to localise specific sites of ER‐membrane fusion.

## RESULTS

### Arabidopsis RTN17 is a member of the clade 6 reticulon protein family

**Figure 1:**
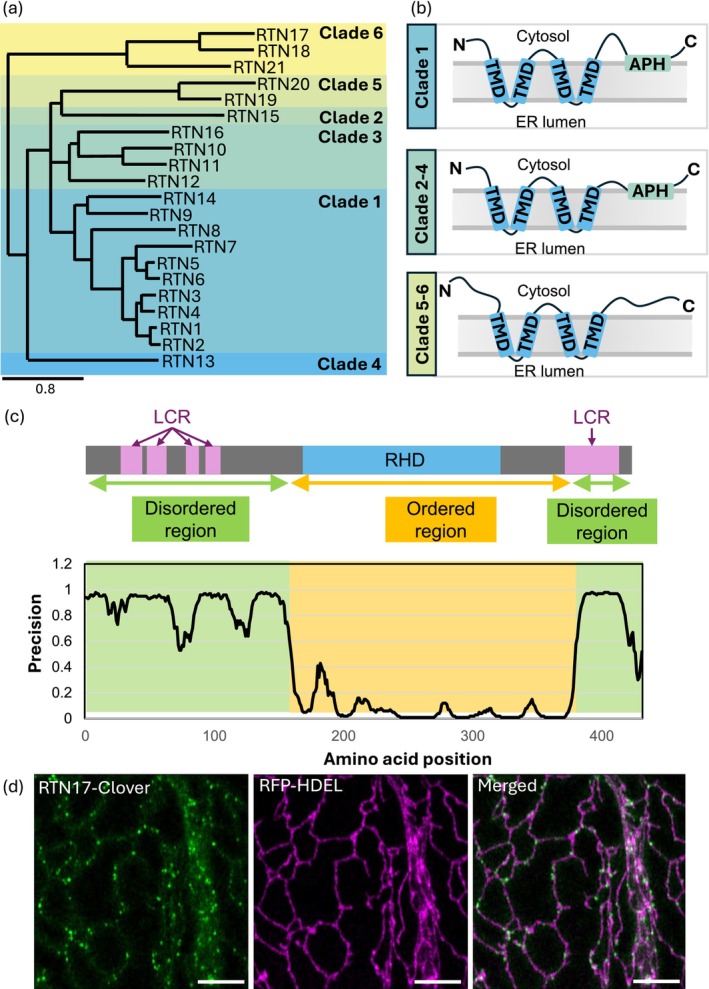
Arabidopsis RTN17 is an ER‐localised member of RTN clade 6 and shows a distinct protein structure characterised by an extended, disordered N‐terminus. (a) Phylogenetic analysis of the 21 reticulon isoforms identified in *Arabidopsis thaliana*. A total of 6 clades of RTNs have been identified, with RTN17 a part of clade 6. (b) Structural characteristics common to RTN protein clades including a W‐shaped arrangement of TMDs and an APH thought to be crucial in the function of RTNs in shaping the ER. The ER membrane is shown in grey. (c) Schematic representation of RTN17 showing the RHD (blue) and low‐complexity regions (LCR, pink). Disordered (green) and ordered (yellow) protein domains are identified by arrows and represented below by a table predicted by DISOPRED3. (d) Representative confocal image of the localisation of RTN17‐Clover (green) and the ER lumenal marker RFP‐HDEL (magenta) when transiently expressed in tobacco epidermal cells. Scale bars = 5 μm.

Arabidopsis RTN17 is part of a larger 21‐member protein family, in which each member can be identified by the presence of a conserved RHD (Nziengui et al., 2007). Based on structural similarities between different members of the RTN protein family and their evolutionary relationships, it is possible to subdivide the RTN protein family into 6 distinct clades (Figure 1a). Clades 1–4 contain a common RHD composed to two transmembrane domain (TMD) wedges that form a W‐shaped structure in the ER membrane (Tolley et al., 2010). This domain is linked by either an extended linker (Clade 1) or short linker (Clade 2–4) to an amphipathic helix (APH). The RHD and APH work in concert to bend ER membranes (Breeze et al., 2016, Brooks et al., 2021, Cui et al., 2011, Jensen et al., 2011), a common feature of RTN proteins. Clade 5–6 are predicted to lack this APH (Brooks et al., 2021) and have extended N‐ and C‐terminal domains (Figure 1b). Predictions by DISOPRED3 have indicated that the extended N‐ and C‐terminal domains of RTN17 are both disordered (Figure 1c). Interrogating the structure of RTN17 with PlaToLoCo also revealed the presence of 5 low‐complexity regions (LCRs), defined as protein domains with unusually limited amino acid diversity (De Pristo et al., 2006). When found together, the presence of large, disordered protein domains containing LCRs are commonly associated with hub proteins (Ekman et al., 2006; Haynes et al., 2006). This is due to the ability of long, simple, flexible linkers to form interactions with multiple protein partners (Chichili et al., 2012; Liu & Huang, 2014). Transient over‐expression of RTN17‐Clover in the model plant expression system *Nicotiana tabacum* (hereafter referred to as tobacco), reveals that RTN17‐Clover localises to distinct puncta that co‐localise with the ER (Figure 1d). RTN17‐Clover shows the same localisation pattern upon stable expression in Arabidopsis (Figure S1).

RTN17 is predicted to produce 3 naturally occurring splice variants. RTN17.1 (hereafter referred to as RTN17) is a full‐length variant, whilst RTN17.2 has a C‐terminal truncation (Δ324‐431aa) and RTN17.3 contains a small deletion near the N‐terminal (Δ144‐156) (Figure S1). All splice variants show similar, punctate ER‐localisation (Figure S1).

### RTN17 shows a typical RTN orientation in the ER membrane

Most RTN proteins have a shared topology in the ER membrane that is essential to their functions as ER shapers (Tolley et al., 2010). Specifically, both the N‐ and C‐terminal domains of the RTN proteins are localised in the cytosol, with the TMDs of the RHD anchoring the protein into the ER membrane. RTN17, however, has a region of hydrophobic residues at approximately 350–375aa, outside of the RHD (Figure S2A). Several TMD prediction algorithms (TOPCONs and OCTOPUS, Tsirigos et al., 2015) suggest this region may form an additional TMD, though this is not supported by Deep TMHMM predictions (Hallgren et al., 2022) (Figure S2B). The presence of this additional TMD would result in the C‐terminus of RTN17 residing in the lumen of the ER.

**Figure 2:**
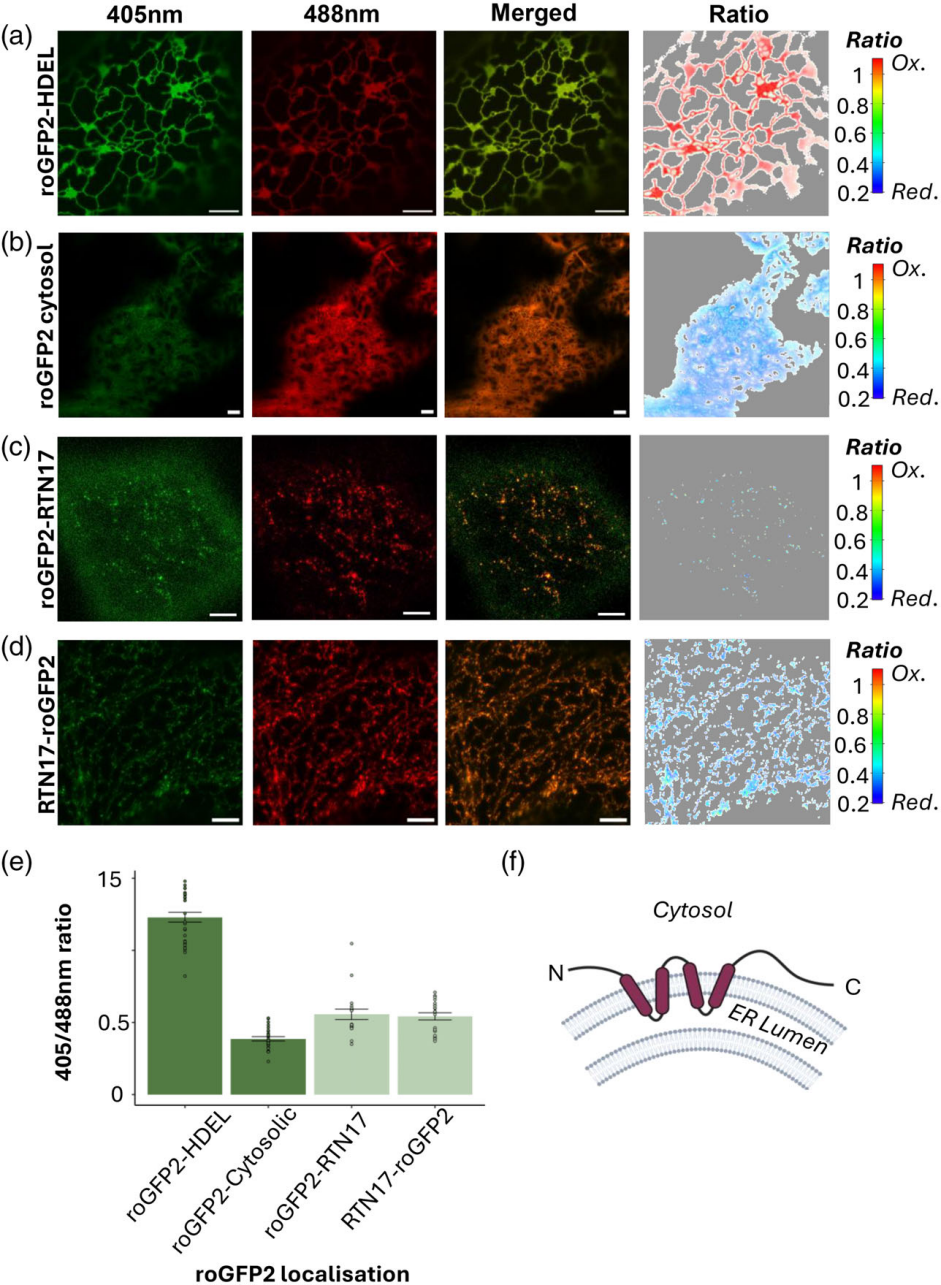
Determination of RTN17 orientation in the ER membrane as using roGFP2. Representative confocal images of tobacco epidermal cells expressing roGFP2 constructs excited by either 405 nm or 488 nm lasers. Also shown are merged images of the two excitations and the intensity ratio between the two channels. Each ratio image is pseudo‐coloured (legend provided) with red indicating an intensity ratio expected in oxidising environments (*Ox*.) and blue representing intensity ratios identified in reducing environments (*Red*.). Representative examples shown for control constructs (a) roGFP2‐HDEL, (b) roGFP2 cytosolic and for the newly developed constructs (c) roGFP2‐RTN17 and (d) RTN17‐roGFP2. Scale bars = 5 μm. (e) Bar graph of the 405/488 nm intensity ratios collected from 3 independent biological replicas, with technical replicas roGFP2‐HDEL *n* = 29, roGFP2 cytosolic *n* = 26, roGFP2‐RTN17 *n* = 19 and RTN17‐roGFP2 *n* = 20. Standard error bars included in diagram. (f) Schematic representation of the membrane topology of RTN17 in the ER.

To non‐invasively probe the membrane topology of RTN17, we used roGFP2, a redox‐sensitive variant of the green fluorescent protein (roGFP2), tagged to each terminus of RTN17. Different plant cellular compartments have different redox states, with the ER possessing an oxidising lumen and the cytosol representing a reducing environment (Brach et al., 2009). roGFP2 contains modified cysteine residues, enabling it to become reduced or oxidised in accordance with its surrounding environment (Brach et al., 2009). The redox environment surrounding roGFP2 affects the disulphide bridges present in roGFP2, in turn affecting the excitation and emission properties of roGFP2 (Hanson et al., 2004). When targeted to an oxidising environment (such as the ER lumen) roGFP2‐HDEL has higher excitation under 405 nm than 488 nm, resulting in an intensity ratio greater than 1 (Figure 2a) (Brach et al., 2009). When targeted to the reducing environment of the cytosol, roGFP2 cytosol has a higher excitation under 488 nm, resulting in a 405/488 nm intensity ratio of less than 1 (Figure 2b). Representative images of both roGFP2‐RTN17 and RTN17‐roGFP2 (Figure 2c,d) demonstrate a 405/488 nm intensity ratio of below 1, more similar to the cytosolic control than the ER lumen control (Figure 2e, Table S1). This similarity of the RTN17 constructs to the cytosolic control as opposed to the ER lumen control suggests a RTN17 membrane topology whereby both termini of RTN17 reside in the cytosol (Figure 2f). There is a significant difference between all measured groups except for N‐ and C‐terminally tagged RTN17 (Kruskal‐Wallis *P*‐value = 2.684 × 10^−15^). However, the difference in the RTN17 tagged roGFP2 intensity is approximately 3 times greater when compared to ER lumen as opposed to the cytosol. This indicates that both termini of RTN17 reside in the cytosol. This membrane topology is the same as for ER shaping RTN proteins in clades 1 and 4 (Sparkes et al., 2010).

### RTN17 does not induce ER membrane bending or constriction

**Figure 3:**
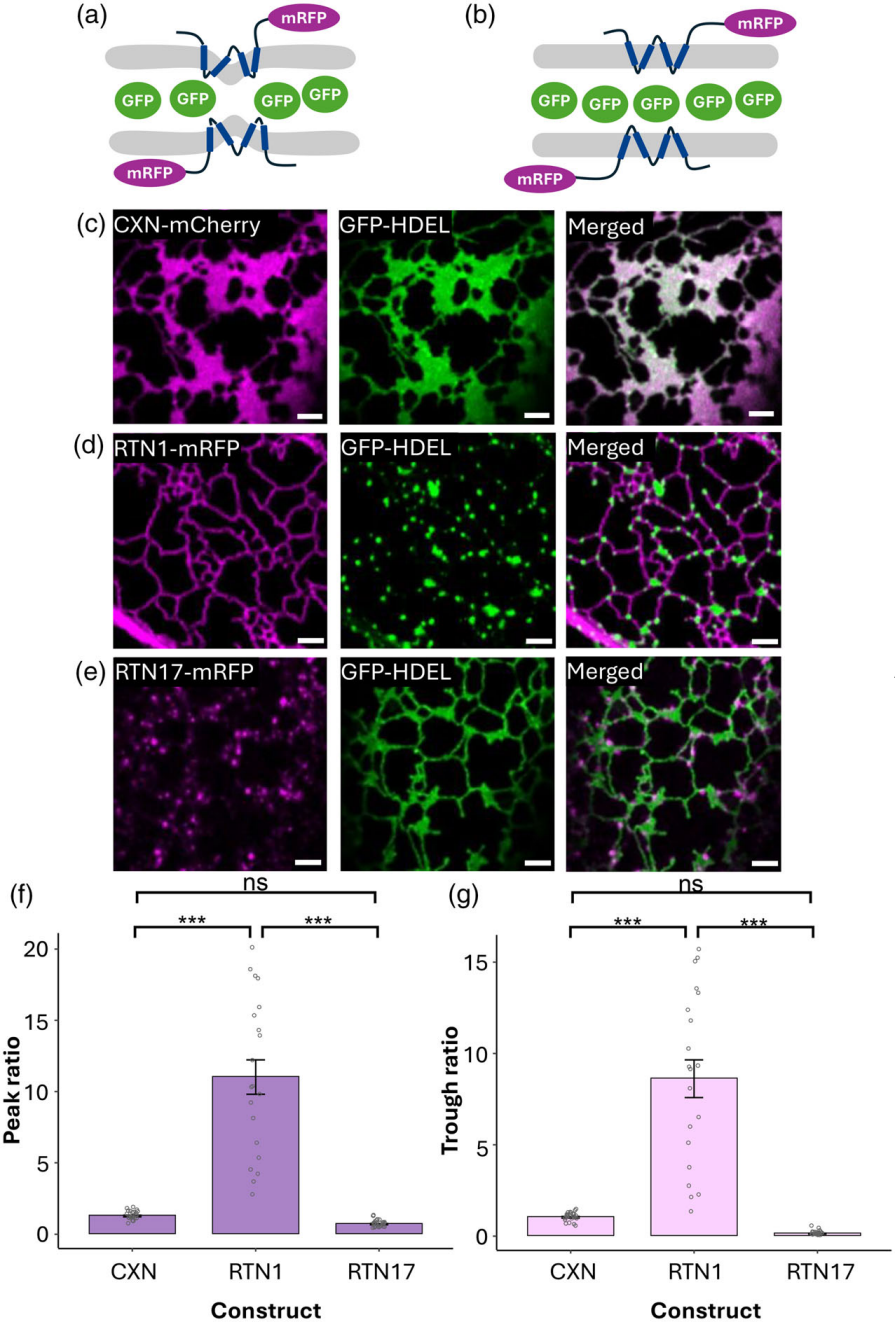
Over‐expression of RTN17 does not induce ER membrane bending as seen on over‐expression of the known ER shaping protein RTN1. Schematic representation of two possible effects of RTN17 over‐expression on ER tubule structure. (a) Represents RTN17 inducing bending of the ER membrane (grey) resulting in exclusion of GFP‐HDEL (and other large ER lumenal contents) from constricted regions of the ER. (b) Represents a case where RTN17 inserts into the ER membrane but does not induce ER bending, allowing for an even distribution of GFP‐HDEL (and other lumenal contents) throughout the ER. Representative confocal images of tobacco epidermal cells after transient over‐expression of the ER lumenal marker GFP‐HDEL (green) and (c) CXN‐mCherry, (d) RTN1‐mRFP and (e) RTN17‐mRFP (magenta). Scale bars = 5 μm, with individual channels and merged images shown. (f) The mean ratio of CXN‐mCherry, RTN1‐mRFP and RTN17‐mRFP intensity peaks compared to GFP‐HDEL intensity peaks as detected along tubules. (g) The mean ratio of intensity troughs alongside individual data points. Non‐statistically significant differences between groups identified using Tukey HSD test after ANOVA are shown by ns, whilst statistically significant differences are denoted by (***) where *P*‐value <0.001. Data collected from *n* = 3 biological replicas and *n* = 25 CXN‐mCherry, *n* = 26 RTN1‐mRFP and *n* = 30 RTN17‐mRFP technical replicas.

RTN17 shows structural and topological similarities to known ER shaping RTN family proteins, including RTN1‐4 (Sparkes et al., 2010). Should RTN17 act as a ‘typical’ ER shaping RTN protein, over‐expression of RTN17 would result in bending of the membrane to create regions of constriction where GFP‐HDEL and other larger ER lumenal contents are excluded from constricted regions of the ER (Figure 3a) (Pain et al., 2019; Sparkes et al., 2010). However, due to the absence of a predicted APH (Brooks et al., 2021), it is also possible that though RTN17 inserts into the ER membrane, it cannot induce membrane bending and ER constriction. In this case, GFP‐HDEL and larger ER lumenal contents would be expected to be spread evenly throughout the ER (Figure 3b). Over‐expression of a non‐shaping ER membrane‐localised truncated protein (CXN‐mCherry) (Groves et al., 2019) does not affect the distribution of GFP‐HDEL throughout the ER lumen (Figure 3c). Over‐expression of the known ER shaper RTN1‐mRFP (Sparkes et al., 2010), however, induces ER constriction to such a point that GFP‐HDEL is restricted to small subregions of the ER lumen, separated by highly constricted regions of ER tubule (Figure 3d). RTN17‐mRFP over‐expression does not appear to overtly affect the distribution of ER lumenal contents (Figure 3e). Using the ER analysis software Analyser (Pain et al., 2019) it is possible to characterise the distribution of the intensity of ER lumenal contents along tubules. Peak ratios represent the maximum fluorescence ratio measured within ER tubules, whereas trough ratios represent the minimum ratio detected within the adjacent cytosolic background region. CXN‐mCherry and RTN17‐mRFP both show a low intensity peak ratio, meaning that peaks in CXN‐mCherry and RTN17‐mRFP do not correlate with peaks in GFP‐HDEL. However, peaks in RTN1‐mRFP correlate with troughs in GFP‐HDEL which is to be expected where constriction forces tubular contents away from areas of tubules with high concentrations of RTN1 (Figure 3f). The same is true of trough ratios where there is a low trough ratio between CXN‐mCherry and RTN17‐mRFP with GFP‐HDEL and RTN1‐mRFP has a high peak ratio (Figure 3g; Table S2). These results indicate that RTN17 is not capable of inducing membrane bending. Together, these findings indicate that RTN17 does not function as a curvature‐generating reticulon, but instead is more consistent with a role in curvature recognition or partitioning to pre‐existing curved ER domains.

### RTN17 preferentially localises to curved ER membranes

**Figure 4:**
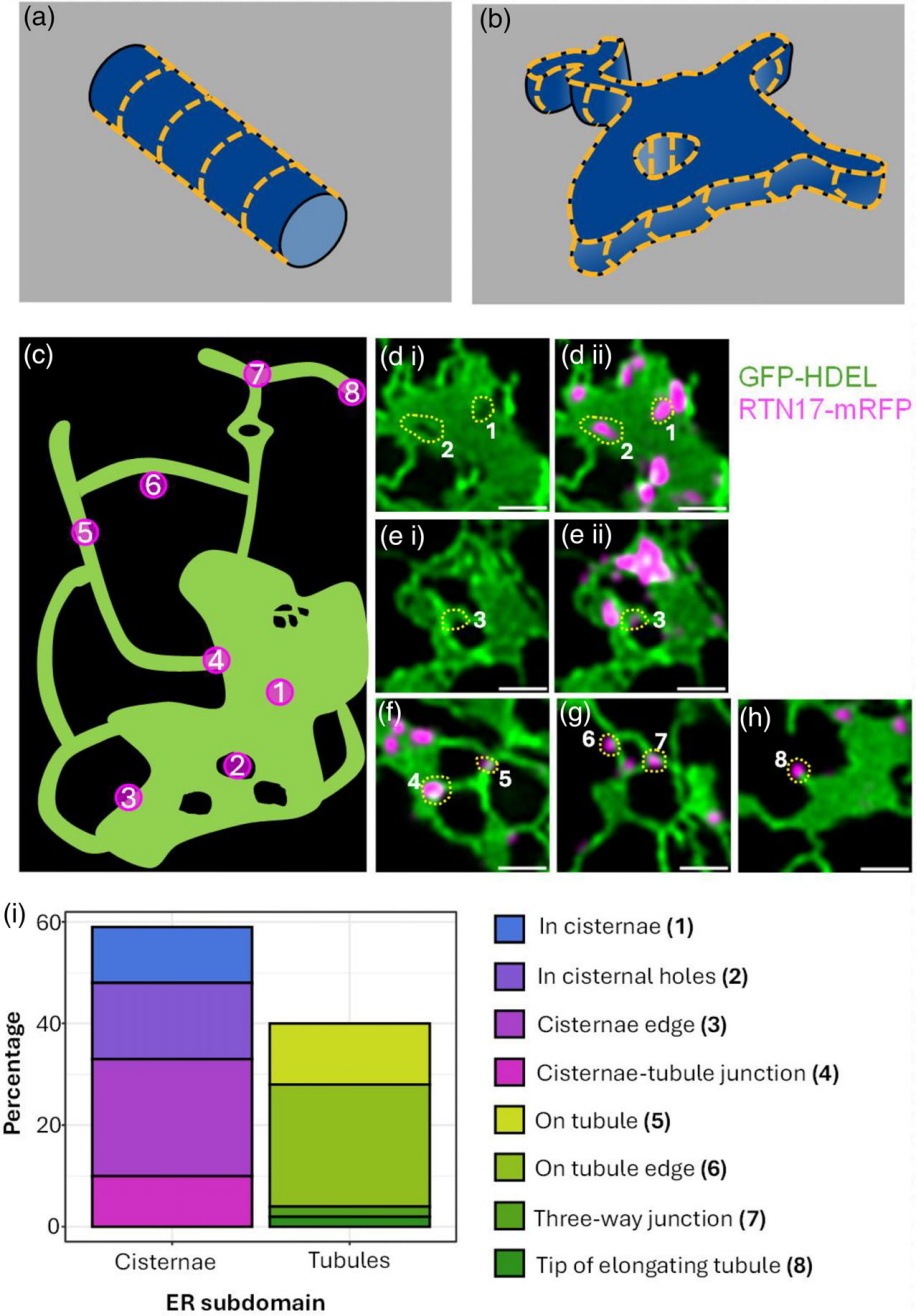
RTN17 preferentially localises to curved ER membranes, in particular tubule and cisternal edges. 3D schematic representation of 2 key ER structural subdomains, (a) tubules and (b) cisternae, with curved membrane surfaces highlighted by dashed yellow lines. (c) Schematic representation of the 8 possible locations of RTN17 puncta alongside representative confocal images of tobacco leaf epidermal RTN17 puncta (magenta) in each position. The ER was identified by transient over‐expression of GFP‐HDEL. (d) Localisation of RTN17‐mRFP puncta (magenta) on (1) cisternae and (2) within cisternal holes, with the GFP‐HDEL (green) channel shown alone (i) and merged with the RTN17‐mRFP channel (ii). (e) RTN17‐mRFP puncta (magenta) localised on the edge of a cisternae (3) shown as a single channel image of GFP‐HDEL (green, i) and merged with RTN17‐mRFP (ii). (f) RTN17‐mRFP (magenta) localised on a cisternae‐tubule junction (4) and on a tubule (5). (g) RTN17‐mRFP puncta localised to tubule edges (6) and on a three‐way tubule junction (7). (h) an RTN17‐mRFP puncta localised to the tip of a growing ER tubule (8). (i) A stacked bar plot of the percentage of RTN17‐mRFP puncta localised to each ER subdomain. Data collected from *n* = 3 biological replicas and *n* = 5 technical replicas. Scale bars = 1 μm.

Though RTN17 is not capable of inducing ER membrane bending, we decided to investigate whether RTN17 may still function on curved ER membranes. The majority of ER membranes are curved (Figure 4a), with only the surface of some ER cisternae being classified as flat (Figure 4b). In order to characterise the localisation of RTN17 puncta across the ER, we subdivided the ER into 8 possible domains (Figure 4c): (1) within cisternae, (2) within holes in cisternae (Figure 4d), (3) on cisternae edges (Figure 4e), (4) at cisternae‐tubule junctions, (5) on tubules (Figure 4f), (6) at tubule edges, (7) at three‐way junctions (Figure 4g), and (8) at the tip of elongating tubules (Figure 4h). We demonstrated that a larger percentage of RTN17‐mRFP puncta localise to cisternae (59%) compared to tubules (41%) (Figure 4i). The majority of RTN17 puncta associated with curved cisternal regions (89%), which here includes cisternal holes, cisternal edges, and cisterna–tubule junctions (subdomains 2–4; Figure 4c–i). This enrichment at curved ER regions supports a model in which RTN17 preferentially associates with membranes of high curvature rather than inducing curvature *de novo*. Similar curvature‐partitioning behaviour has been described for membrane‐associated proteins that lack strong membrane‐wedging elements but selectively accumulate on curved surfaces (Jensen et al., 2011).

### RTN17 modulates RHD3 localisation and together they influence the structure and dynamics of the ER

**Figure 5:**
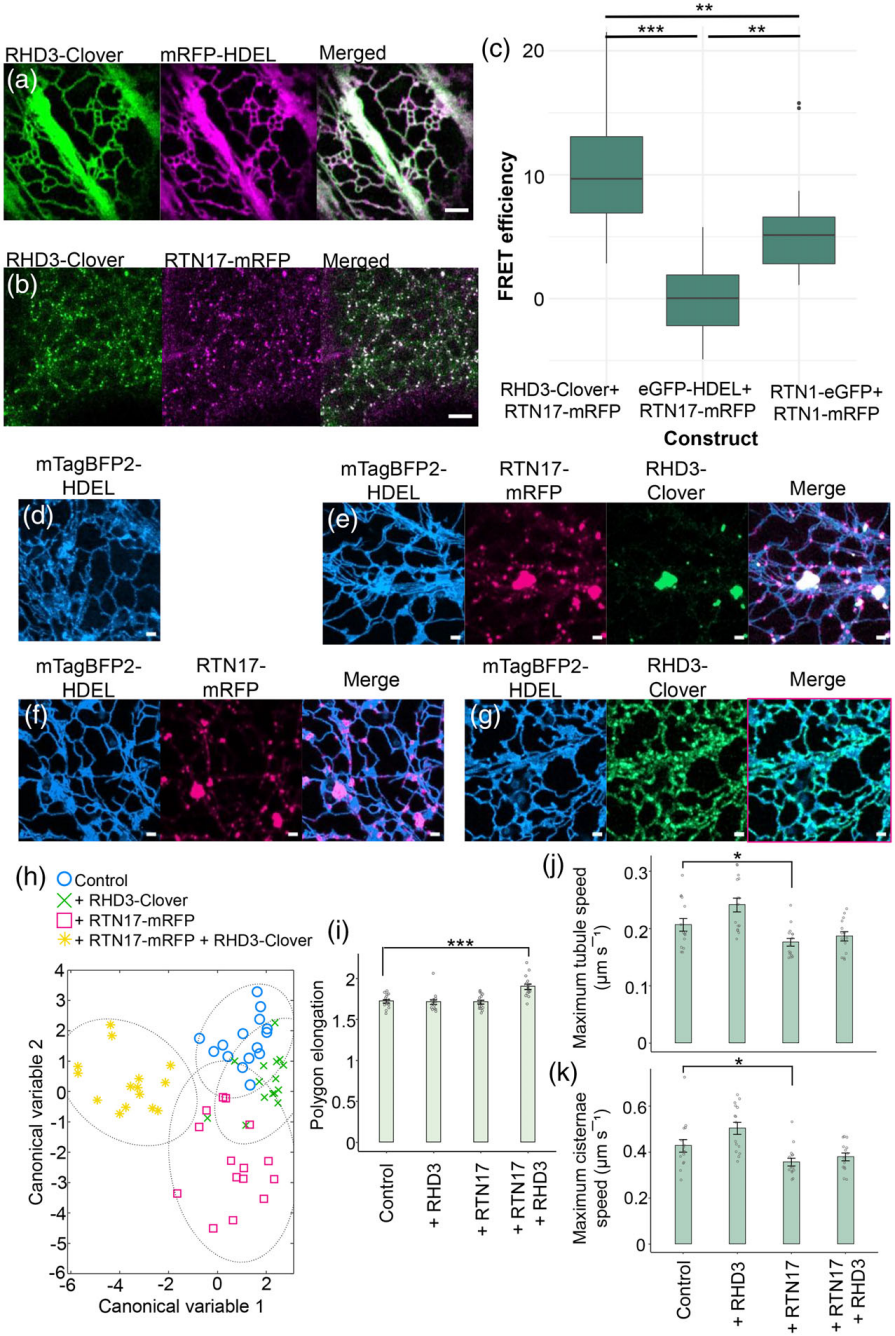
RTN17 physically interacts with RHD3 to reshape the plant ER. Over‐expression of (a) RHD3‐Clover (green) alongside the ER marker mRFP‐HDEL (magenta) with the merged image shown. (b) Representative images of over‐expression of RHD3‐Clover (green), alongside RTN17‐mRFP (magenta) with the merged image shown. Scale bars = 5 μm. (c) Boxplot showing the FRET efficiencies of RHD3‐Clover and RTN17‐mRFP, the negative control GFP‐HDEL and RTN17‐mRFP and the positive control RTN1‐eGFP and RTN1‐mRFP. Results taken from at least 3 biological repeats and technical replicas RHD3‐Clover + RTN17‐mRFP *n* = 83, GFP‐HDEL + RTN17‐mRFP *n* = 10, and RTN1‐eGFP + RTN1‐mRFP *n* = 14. There is a significant difference between the FRET efficiency of all groups (ANOVA *P*‐value = 9.87 × 10^−11^, Tukey test *P*‐values: RHD3‐Clover + RTN17‐mRFP/GFP‐HDEL + RTN17‐mRFP *P* < 1.00 × 10^−6^; RTN1‐eGFP + RTN1‐mRFP/GFP‐HDEL + RTN17‐mRFP *P* = 2.81 × 10^−3^; RTN1‐eGFP + RTN1‐mRFP/RHD3‐Clover + RTN17‐mRFP *P* = 2.36 × 10^−3^). Representative images of the structure of the ER of tobacco epidermal cells shown in (d) mTagBFP2‐HDEL (blue) after over‐expression of (e) RTN17‐mRFP (magenta) and RHD3‐Clover (green) together and with (f) RTN17‐mRFP and (g) RHD3‐Clover alone. Scale bars = 1 μm. (h) Pairwise scatter plots of the first 2 significant canonical variables from the MANOVA analysis grouped by treatment. The dotted line around each group represents the 95% confidence limit. Bar plots with standard error bars showing the (i) mean polygon elongation, (j) maximum tubule speed (μm s^−1^) and (k) maximum cisternae speed (μm s^−1^) alongside individual points. Only significant comparisons between test groups and the control (mTagBFP2‐HDEL) are shown. Statistical analysis for (i–k): ANOVA, *P*‐value = 1.3 × 10^−7^, 6.13 × 10^−5^ and 1.88 × 10^−4^, Tukey post hoc test *P*‐value = 1.70 × 10^−5^, 4.25 × 10^−2^ and 4.61 × 10^−2^ for polygons, tubules and cisternae, respectively, *P*‐values adjusted with Bonferroni correction, +1.1%, −16.5% and −17.3% change, respectively. Results taken from at least 3 biological replicas, for structure analysis technical replicas control *n* = 15, RHD3 *n* = 15, RTN17 *n* = 15, and RTN17 + RHD3 *n* = 15, and for ER dynamic analysis technical replicas control *n* = 15, RHD3 *n* = 15, RTN17 *n* = 15, and RTN17 + RHD3 *n* = 14. Significant results are denoted by *P‐value 0.05–0.001, **P‐value 0.001–0.001 and ***P‐value ≤0.001. Full statistical analysis for H‐K are presented in Table S3.

Reticulon proteins in other species have previously been shown to interact with the ER fusogen atlastin (homology to the plant RHD3). Interactions between reticulon proteins and RHD3 have previously been reported in plants. For example, RTN proteins have been shown to physically associate with RHD3 and influence ER morphology (Lee et al., 2013), consistent with observations in yeast and mammalian systems where reticulons cooperate with atlastins to regulate ER network formation (Hu et al., 2009; Wang et al., 2016). These findings provide a strong rationale to investigate whether RTN17 similarly interacts with RHD3. This interaction has been shown to be essential to the maintenance of normal ER morphology (Wang et al., 2016); therefore, we decided to test whether Arabidopsis RTN17 may interact with RHD3 *in vivo*. Firstly, transient over‐expression of RHD3‐Clover and RTN17‐mRFP shows that RTN17‐mRFP over‐expression alters the localisation of RHD3‐Clover throughout the ER (Figure 5a,b). When over‐expressed with the insensitive RFP‐HDEL, RHD3‐Clover is spread evenly throughout the ER (Figure 5a). However, when co‐expressed with RTN17‐mRFP, RHD3‐Clover overlaps with RTN17‐mRFP into foci that are distributed throughout the ER (Figure 5b). To establish whether this re‐localisation is caused by a physical interaction between RHD3 and RTN17, we used AP‐FRET (Acceptor photobleaching– Förster resonance energy transfer). Here an increased signal in the donor fluorophore occurs in response to bleaching of the acceptor fluorophore, if the two are within close enough proximity indicating protein–protein interaction (Truong & Ikura, 2001). As a positive control, AP‐FRET was performed with co‐expression of eGFP‐RTN1 and mRFP‐RTN1; this protein interaction has been previously confirmed to interact via FRET‐FLIM (Kriechbaumer, Botchway, et al., 2015) and shows a FRET efficiency of 5.99 ± 1.60%. The negative control RTN17.1‐mRFP with GFP‐HDEL displayed a FRET efficiency of 0.08 ± 0.03%. AP‐FRET with RHD3‐Clover and RTN17.1‐mRFP resulted in a high FRET efficiency of 10.19 ± 1.12% (Figure 5c), indicating a physical interaction between these two proteins.

As one of the primary functions of RHD3 is maintaining the structure of the ER through homotypic membrane fusion (Wang et al., 2016), we tested whether over‐expression of RTN17 and RHD3 (Figure 5d,f,g) either alone or in combination is sufficient to modify ER architecture (Figure 5e). To distinguish the effects of RTN17‐RHD3 interaction from downstream structural consequences, we quantified ER organisation using multiple ER metrics, referring to a MANOVA comparison of all measured ER metrics including polygon area, polygon elongation, tubule length, and tortuosity (Table S3; Pain et al., 2019).

This analysis revealed that over‐expression of RTN17‐mRFP results in significant alterations to ER structure, and that these changes are distinct from those observed upon co‐expression of RTN17‐mRFP and RHD3‐Clover (Figure 5h). In contrast, over‐expression of RHD3‐Clover alone produces only minimal changes relative to the control ER (Figure 5h). This is consistent with previous reports that RHD3 primarily regulates ER connectivity rather than global ER morphology (Chen et al., 2011; Zhang, Wu, et al., 2013). A pairwise scatter plot of the first two canonical variables (composite variables created through canonical correlation analysis to summarise the relationship between two sets of variables) shows RTN17‐mRFP alone and with RHD3‐Clover have the greatest effect on ER structure (MANOVA Pillai's trace *P*‐value = 7.91 × 10^−7^ and Roy's largest root *P*‐value = 2.76 × 10^−7^). Of particular interest is that polygonal regions show a significant increase in elongation (Figure 5i) on RTN17 and RHD3 over‐expression (ANOVA, *P*‐value = 1.3 × 10^−7^, Tukey post hoc test 1.70 × 10^−5^, *P*‐values adjusted using a Bonferroni correction, +1.1%, Figure 5i) whilst tubule tortuosity is significantly reduced (Table S3; ANOVA, *P*‐value = 4.81 × 10^−2^, Tukey post hoc test 2.04 × 10^−2^, *P*‐values adjusted with Bonferroni correction, −0.25%). Together, these changes indicate that the ER network becomes straighter and more ordered. Increased polygon elongation and reduced tortuosity are consistent with cytoskeletal guidance, where ER tubules align along linear tracks such as microtubules, resulting in reduced tortuosity and increased anisotropy (Hamada et al., 2014). These observations suggest that RTN17, particularly in combination with RHD3, promotes alignment of the ER with the underlying cytoskeleton.

Similarly, loss of RTN17 expression in Arabidopsis results in minimal detectable changes in overall ER structure (Figure S3A,B). Small nodules (0.3–0.4 μm^2^) can be observed along ER tubules (Figure S3C). Although this increase is not statistically significant (Kruskal‐Wallis *P*‐value = 0.602), similar structures have previously been reported following treatment with oryzalin (Langhans et al., 2009), a drug that inhibits the polymerisation of microtubules (Morejohn et al., 1987). Notably, such nodules are not always reproducible and may reflect indirect effects such as ER stress at high drug concentrations (Sparkes et al., 2009), and should therefore be interpreted cautiously. Nevertheless, their presence is consistent with disruption of ER–microtubule interactions, suggesting that loss of RTN17 may perturb this relationship. In addition, loss of RTN17 also results in significantly longer ER tubules (ANOVA *P*‐value = 0.039, Figure S3D), which may reflect reduced microtubule‐guided tubule extension or impaired localisation of RHD3 to sites of junction formation. Stable over‐expression of RTN17‐Clover in *Arabidopsis thaliana* did not result in significant changes to ER structure (Figure S4), and neither stable over‐expression nor knockout of RTN17 produces gross morphological or developmental phenotypes.

Further analysis revealed that RTN17 over‐expression alone significantly affects the dynamics of the ER. Specifically, the maximum speed of both ER tubules and cisternae is significantly decreased upon RTN17‐mRFP over‐expression (Figure 5j,k, Figure S5). In contrast, expression of RHD3 alone leads to a slight increase in both cisternal and tubular maximum speeds, with a significantly higher mean cisternal speed (Table S3). Co‐expression of RTN17 and RHD3 results in overall non‐significative changes in ER movement, except for mean cisternal persistency, which was significantly increased (Table S3). These findings suggest that RTN17 modulates ER dynamics, potentially by stabilising ER–cytoskeleton interactions.

### RTN17 links curved ER membranes to the microtubule cytoskeleton

**Figure 6:**
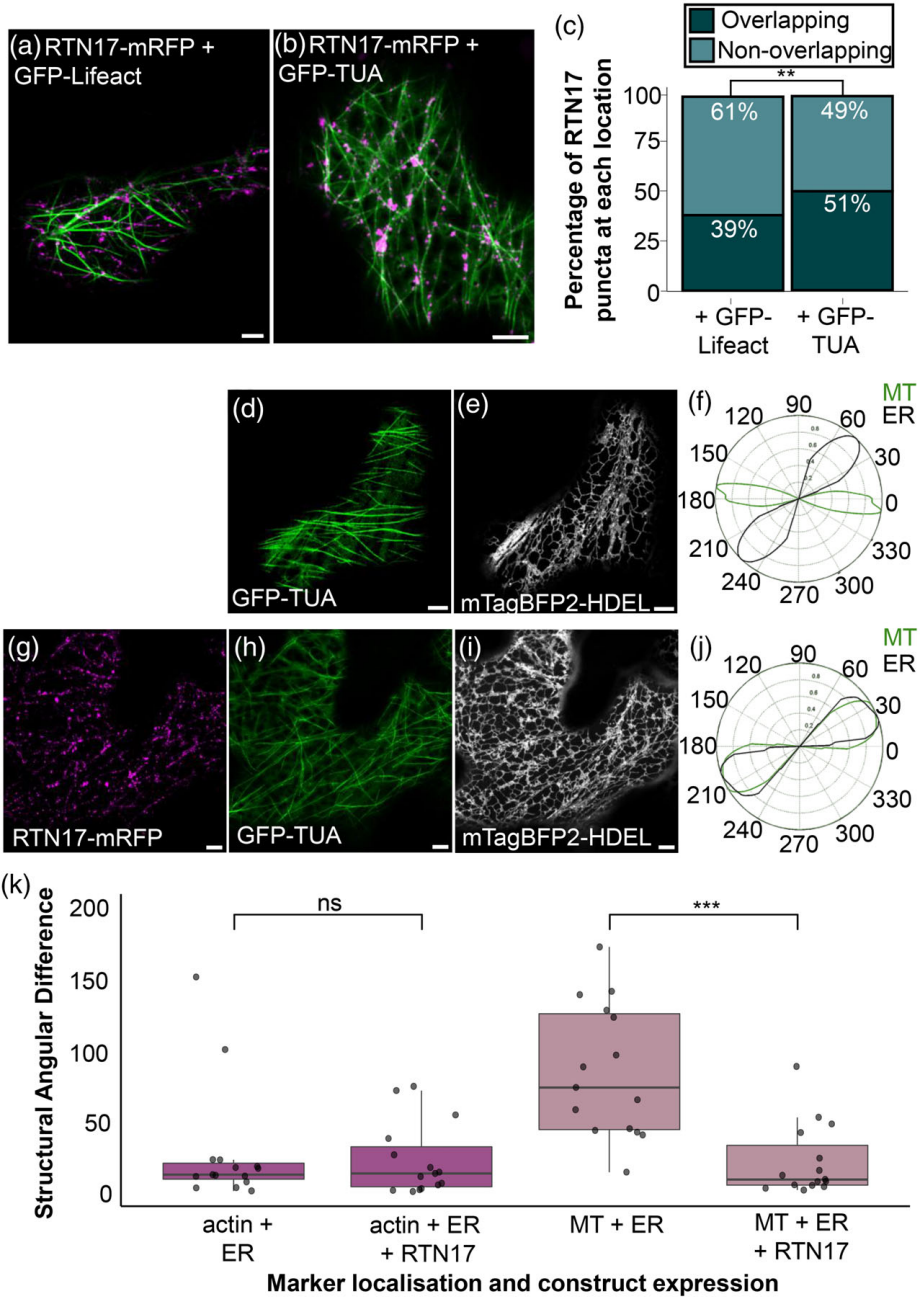
RTN17 puncta preferentially overlap with microtubules and alter the orientation of the ER to match the orientation of microtubules. Representative images of tobacco leaf epidermal cells transiently expressing the actin marker (a) GFP‐Lifeact (green) + RTN17‐mRFP (magenta). Representative images of the microtubule marker (b) GFP‐TUA (green) + RTN17‐mRFP (magenta). (c) Stacked boxplot comparing the percentage of RTN17‐mRFP puncta overlapped with both the actin cytoskeleton and microtubules. RTN17 exhibits a significant preference for microtubules (**, Student's *t*‐test *P*‐value = 0.005). Representative images of tobacco leaf epidermal cells transiently expressing (d) GFP‐TUA and (e) the ER marker mTagBFP2‐HDEL. (f) The mean orientation of ER tubules (black) and microtubules (MT, green) in the displayed cell. Representative images of (g) RTN17‐mRFP, (h) GFP‐TUA, and (i) mTagBFP2‐HDEL alongside (j) the mean orientation of ER tubules (black) and microtubules (MT, green) in the displayed cell. (k) The mean angular difference between the actin and ER, marked with GFP‐Lifeact and mTagBFP2‐HDEL, with and without co‐expression of RTN17‐mRFP and the mean angular difference between microtubules, marked with GFP‐TUA, alongside the ER marked with mTagBFP2‐HDEL, with and without RTN17‐mRFP. Actin‐ER shows no significant angular difference (ns, Welch's *t*‐test *P*‐value = 0.70) but microtubules‐ER has a significant reduction in the mean angular difference (***, Welch's *t*‐test *P*‐value = 1.49 × 10^−4^). Data collected from at least *n* = 3 biological replicas; (c) GFP‐Lifeact *n* = 30, and GFP‐TUA *n* = 30 technical replicas and (k) *n* = 15, actin + ER; *n* = 15, RTN17 + actin + ER; *n* = 15, microtubules (MT) + ER; *n* = 15, RTN17 + MT + ER technical replicas. Scale bars = 5 μm.

The changes of ER structure and dynamics suggest that RTN17 may mediate an interaction with either the actin or microtubule cytoskeleton. To examine this possibility, we measured the proportion of RTN17‐mRFP foci that overlapped with the actin cytoskeleton (marked with GFP‐Lifeact, Figure 6a) compared to overlap with the microtubule cytoskeleton (marked with GFP‐TUA, Figure 6b). 39% of RTN17‐mRFP puncta were found to overlap with the actin cytoskeleton, whilst 51% overlapped with the microtubule cytoskeleton (Figure 6c). Due to the presence of the large vacuole, a large proportion of the cellular substructure, in particular membrane‐bound organelles and the cytoskeleton, is forced into a thin layer between the vacuole and the plasma membrane. Therefore, overlap between RTN17‐mRFP puncta and both cytoskeletons is expected; however, the increase in puncta associated with the microtubule cytoskeleton is significant.

Using CytoSpectre, a software designed to analyse the orientation of features in micrographs, the ER of tobacco epidermal cells does not typically fully align with the microtubule cytoskeleton (Figure 6d–f,k). However, over‐expression of RTN17‐mRFP alters the structure of the ER so that it aligns much more extensively with the microtubule cytoskeleton (Figure 6g–k). We also investigated whether RTN17 over‐expression causes the ER to conform more towards the actin cytoskeleton; however, we found no significant difference in the mean actin‐ER angular difference with and without RTN17 over‐expression (Figure 6k). This is unlike comparing the effect of RTN17 on the ER and microtubule cytoskeleton, that shows a significant reduction in the mean angular difference of the ER and microtubules (Figure 6k).

To further assess whether the association between RTN17 and microtubules reflects a functional interaction rather than a consequence of cytoskeletal abundance, we examined ER dynamics following microtubule depolymerisation with oryzalin (Figure S6). Disruption of microtubules resulted in significant changes in ER dynamics (MANOVA; oryzalin: Pillai's trace *P*‐value = 7.12 × 10^−3^), with overall increased ER motility compared with untreated groups (Figure S6), demonstrating that microtubules contribute to the stabilisation of the ER network. In contrast, RTN17 over‐expression reduced ER dynamics (MANOVA; RTN17: Pillai's trace *P*‐value = 3.51 × 10^−3^), consistent with a stabilising effect on the network (Figure S6; Figure 5j,k). Depolymerisation of microtubules partially reversed this reduction in ER dynamics induced by RTN17, with ER speeds increasing relative to untreated RTN17 over‐expressing cells (Figure S6). As microtubule depolymerisation was not always complete and residual microtubule structures remained visible in some cells (Figure S6), the extent of this recovery may be underestimated. Nevertheless, the partial rescue observed, together with the significant RTN17‐oryzalin interaction (MANOVA; RTN17 × oryzalin: Pillai's trace *P*‐value = 1.23 × 10^−2^), supports the hypothesis that the effect of RTN17 on ER dynamics is at least partially dependent on the microtubule cytoskeleton.

Together with the preferential alignment of the ER along microtubules (Figure 6g–k), these findings suggest that RTN17‐mediated regulation of ER dynamics requires, at least in part, an intact microtubule network. Collectively, these data provide functional evidence that RTN17 contributes to ER–microtubule coupling rather than simply co‐localising with abundant cortical microtubules.

## DISCUSSION

### RTN17 is an atypical RTN protein family member

Our data shows that RTN17, identified as a member of the reticulon proteins family through its conserved RHD, has an unusual protein structure. Firstly, it harbours extended N‐ and C‐terminal domains and lacks the predicted amphipathic helix found in many reticulons (Breeze et al., 2016; Brooks et al., 2021; Cui et al., 2011; Jensen et al., 2011). In addition, these extended terminal regions include substantial intrinsically disordered segments, enriched in low‐complexity regions (LCRs)—features often associated with hub proteins capable of multiple protein–protein interactions (Ekman et al., 2006; Haynes et al., 2006). These characteristics suggest that RTN17 may act as a multifunctional scaffold rather than purely as a curvature‐generating reticulon.

Other members of the Arabidopsis reticulon family have been shown to interact with RHD3, including RTN3 and RTN6, supporting a broader functional relationship between reticulons and ER fusion machinery (Kriechbaumer, Seo, et al., 2015; Lee et al., 2013). However, RTN family members display diverse functional behaviours. For example, RTN20 has been reported to localise to punctate ER structures but does not induce membrane constriction and has instead been linked to roles in lipid metabolism and sterol biosynthesis (Kriechbaumer, Maneta‐Peyret, et al., 2018). Similarly, RTN19 shows a more homogeneous ER distribution and has been associated with enzymatic functions in sterol or lipid biosynthetic pathways rather than direct membrane shaping (Kriechbaumer, Breeze, et al., 2018; Kriechbaumer, Maneta‐Peyret, et al., 2018). In addition, several plant RTN proteins have been implicated in plasmodesmata structure and function, where they contribute to the formation and maintenance of highly curved ER membranes within desmotubules, as well as viral protein movement (Knox et al., 2015; Kriechbaumer, Botchway, et al., 2015; Tilsner & Kriechbaumer, 2022). Unlike these proteins, RTN17 not only forms punctate structures but also induces measurable changes in ER organisation, particularly when co‐expressed with RHD3, suggesting a role that integrates membrane organisation with spatial regulation of ER fusion. Despite these atypical features, RTN17 exhibits the classical reticulon arrangement within the ER membrane (Tolley et al., 2010): both N‐ and C‐termini face the cytosol, and the predicted hydrophobic region in its extended C‐terminal domain does not appear to function as a TMD. This suggests that the hydrophobic stretch likely reflects a membrane‐adjacent or membrane‐associated region, rather than a full spanning TMD. Functionally, however, RTN17 does not promote ER constriction in over‐expression, in contrast to other well‐characterised ER‐shaping reticulons (Breeze et al., 2016). This divergence implies that RTN17 serves a distinct functional role.

### Localization and interaction with the ER fusion machinery

In localisation studies, RTN17 is found in punctate structures decorating the ER network, preferentially on curved membrane surfaces. Notably, RTN17 physically interacts with the ER‐shaping GTPase RHD3 (Chen et al., 2011; Zhang, Mallery, et al., 2013) (confirmed via AP‐FRET). Beyond mere binding, RTN17 actively relocalises RHD3 to the RTN17‐defined puncta, one of the clearest examples of a reticulon influencing RHD3 localisation *in planta*, consistent with previous reports of RTN–RHD3 interactions (Lee et al., 2013). This re‐localisation supports a model in which RTN17 not only binds RHD3, but also sequesters or organises it at specific curved ER domains.

Given that RHD3 is implicated in homotypic ER membrane fusion, the RTN17–RHD3 interaction suggests that RTN17 may play a regulatory role in fusion events, particularly at curved ER regions. By recruiting RHD3 to defined puncta, RTN17 could create microdomains where fusion is promoted or modulated.

RTNs are known to oligomerise within the membrane, and their asymmetric distribution can influence local membrane curvature (Hu et al., 2008; Voeltz et al., 2006). One possible mechanism is that local enrichment of RTNs within one leaflet of the bilayer generates differential surface area, leading to bending. In this context, RTN17 puncta may represent sites of reticulon clustering that modulate curvature indirectly, not by inducing tubules, but by stabilising or redirecting existing curvature. In principle, different physical mechanisms would predict distinct curvature outcomes: local expansion of one leaflet would favour convex curvature, whereas restriction or crowding effects could favour concave curvature. Mechanisms based on leaflet expansion would be expected to preferentially generate convex curvature. However, given the absence of an amphipathic helix and the lack of a constriction phenotype, RTN17 is unlikely to generate curvature via leaflet asymmetry, and instead more plausibly partitions into pre‐existing curved ER domains. This interpretation is consistent with our experimental observations showing no membrane constriction upon RTN17 over‐expression.

### Interplay with the cytoskeleton and implications for ER dynamics

Beyond the reticulon–RHD3 axis, RTN17 over‐expression leads to distinct changes in the ER network and its relationship with the cytoskeleton. This interpretation is supported by the observed increase in polygon elongation and reduction in tubule tortuosity, both indicative of ER alignment along linear cytoskeletal tracks. Specifically, over‐expressing RTN17 alters ER morphology in a manner correlated with changes in microtubule architecture: the ER aligns with underlying microtubules, rather than actin, which is unusual for an ER‐localised protein. This suggests a microtubule‐dependent scaffolding role for RTN17. In fact, the phenotype is reminiscent of the function of the REEP family in metazoans (for which no clear Arabidopsis homologue exists) — proteins that bridge ER tubules to the microtubule cytoskeleton (Shibata et al., 2024; Yalçın et al., 2017).

Importantly, RTN17 over‐expression also reduces the maximum speed of ER membrane remodelling (Figure 5j,k), consistent with the idea that RTN17 may stabilise rather than simply allow dynamic remodelling of the ER network. We thus propose that RTN17 acts as a stabilised scaffold: by linking ER tubules to microtubules and recruiting RHD3 to these sites, RTN17 establishes fusion‐competent domains at appropriately curved ER surfaces, thereby modulating both ER network architecture and dynamics.

### Proposed model

**Figure 7:**
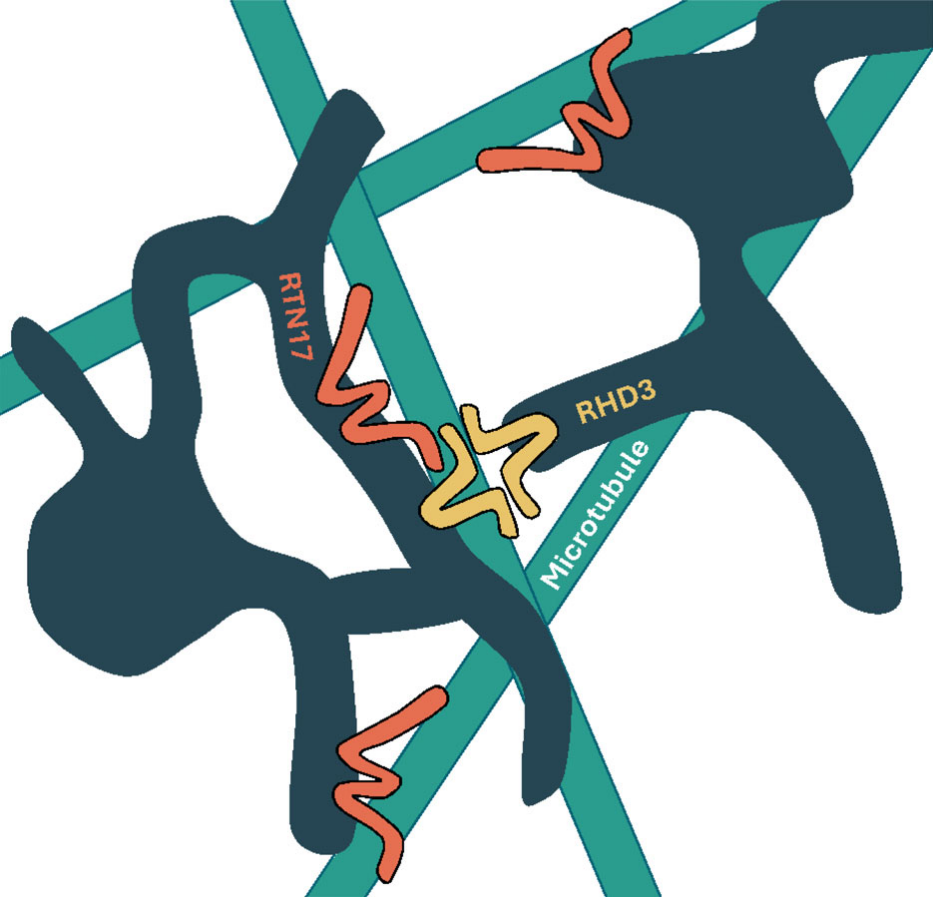
Summary of the function of RTN17 in plant cells. RTN17 (orange) is localised to the plant ER (dark green) on curved surfaces, with both termini facing the cytosol. RTN17 interacts with RHD3 (yellow) to localise RHD3 to the underlying microtubule cytoskeleton (green). Here RHD3 can complete its function as an ER fusogen.

In summary, we propose a model in which RTN17 defines specialised ER microdomains at curved surfaces, where it recruits RHD3 and connects the ER to the microtubule cytoskeleton. Within these domains, RTN17 may stabilise nascent fusion sites, and by doing so, modulate the rate and spatial pattern of ER fusion rather than driving large‐scale tubulation or constriction. One possibility is that asymmetric accumulation of RTN17 and its associated proteins on one side of ER tubules generates local curvature biases, influencing tubule directionality. These sites may also correspond to ER‐cytoskeleton anchoring points or membrane contact sites, stabilising ER structure and reducing membrane mobility. In this way, RTN17 acts as a multifunctional hub: integrating membrane curvature, cytoskeletal anchoring, and fusion machinery recruitment to fine‐tune ER network formation and stability (Figure 7).

## METHODS

### Bioinformatics

Protein and nucleotide sequence information for the gene of interest was acquired from The Arabidopsis Information Resource (TAIR, arabidopsis.org) database (Berardini et al., 2015), with the accession number of At2g20590 for RTN17. Phylogenetic trees were produced using phylogeny.fr (Dereeper et al., 2008) one‐click analysis. Disordered regions were identified using DISOPRED3 (Jones & Cozzetto, 2015), whilst LCR were identified using PlaToLoCo (Jarnot et al., 2020).

Hydropathicity calculated using the technique proposed by Kyte and Doolittle (1982) as implemented by Expasy (Gasteiger et al., 2003). Window size set to 12 to highlight predicted TMDs. Predictions of TMDs produced using TOPCONS (Tsirigos et al., 2015) and Deep TMHMM (Hallgren et al., 2022) using standard settings.

### Expression constructs

Genes of interest were cloned using Gateway technology (Invitrogen). Primers were obtained from Eurofins Genomics and all PCRs were performed using Q5 high‐fidelity DNA polymerase (New England Biolabs). All constructs were cloned for expression under the cauliflower mosaic virus (CaMV) promoter (Wang & Bai, 1990) and various fluorophores. Numerous previously published plant expression constructs were also used as a part of this work (details in Table S4).

### Plant tissues and construct expressions

Transient expression of constructs in tobacco epidermal cells was performed using Agrobacterium‐mediated infiltrations according (Sparkes et al., 2006). To describe the method briefly: *Nicotiana tabacum* (SR1 cv Petit Havana) is grown under greenhouse conditions at 23°C with a 12‐h light/dark cycle until 4–6 weeks old. Transformed *Agrobacterium tumefaciens* strain GV3101 is grown overnight at 28°C with shaking and then pelleted by centrifugation at 2200 × ***g*** at room temperature. The pellet is then washed, re‐centrifuged and resuspended in freshly made infiltration buffer (5 mg ml^−1^ glucose, 50 mm MES, 2 mm Na_3_PO_4_·12H_2_O and 0.1 mm acetosyringone in deionised water). The bacterial suspension is then diluted to an appropriate optical density (OD_600_ = 0.1) and then injected into the abaxial side of a fully expanded tobacco leaf with a syringe without a needle. OD_600_ = 0.1 was used for all infiltrations (single and multiple), except for the co‐expressions with mTagBFP2‐HDEL; here an OD_600_ = 0.05 was applied for mTagBFP2‐HDEL. Infiltrated plants are then placed in an incubator for 2 days at 23°C with a 12‐h light/dark cycle prior to imaging.

Stable expression of constructs in *Arabidopsis thaliana* (Columbia‐0) is achieved using the floral dip method as described in Sparkes et al. (2006). Briefly: transformed Agrobacterium (GV3101) are grown as part of a large (100 mL) culture overnight at 28°C with shaking. The culture is then pelleted and resuspended in floral dip medium (5% sucrose and 500 μl L^−1^ Silwet L77 in deionised water). Flowering Arabidopsis stems are then dipped into this solution and gently agitated for approximately 30 seconds. Plants are then wrapped in clingfilm for 24 h to promote survival and penetration of Agrobacterium. After plant growth and seed harvesting, seeds were selected on silica dioxide plates watered with 5 μg ml^−1^ glufosinate‐ammonium in sterile ¼ concentration Murashige and Skoog medium in deionised water (Davis et al., 2009). Prior to growth for imaging, seeds were sterilised for 3 mins in 70% ethanol with minor agitation. Seeds were then dried fully and transferred to ½ concentration MS agar plates. Plates were incubated in the dark at 4°C for 3 days prior to growth in a plant incubator at 21°C for a further 10 days.

### Imaging parameters

Leaf sections (~5 mm^2^) from Agrobacterium‐infiltrated *Nicotiana tabacum* were excised with a sharp razor blade and immediately mounted in water for microscopy. For microtubule depolymerisation, samples were treated with 10 μm oryzalin (Oryzalin Technical Min 95%, DowElanco, dissolved in DMSO) for 45 min prior to imaging. Control samples were treated with DMSO alone at a final concentration of 1 μl ml^−1^.

Epidermal cells were visualised using a Zeiss LSM 880 confocal microscope equipped with an Airyscan detector, employing either a Zeiss Plan‐Apochromat 100×/1.46 NA oil‐immersion objective or a Plan‐Apochromat 63×/1.46 NA oil‐immersion objective. Enhanced green fluorescent protein (eGFP) and Clover were excited at 488 nm, and emission was collected at ~523 nm; monomeric red fluorescent protein (mRFP) was excited at 561 nm, with emission detected at ~579 nm; mTagBFP2 was excited at 405 nm with emission detected at 450 nm. All images were acquired in line‐switch scanning mode with 2–4× frame averaging to enhance signal quality.

### RTN17 membrane topology analysis

Fusion constructs comprising roGFP2‐RTN17 and RTN17‐roGFP2 were generated via Gateway cloning into the pCMO1 and pSS01 destination vectors to achieve N‐ and C‐terminal fusions, respectively (Brach et al., 2009). These constructs were agroinfiltrated into *Nicotiana tabacum* leaves and imaged 2 days post‐infiltration. Leaf samples were visualised using a Zeiss LSM 880 confocal microscope equipped with an Airyscan detector and a Plan‐Apochromat 63×/1.46 NA oil‐immersion objective. Fluorescence from roGFP2 was elicited using both 405 nm (oxidised form) and 488 nm (reduced form) laser lines, with emission collected within the 500–555 nm range. Autofluorescence was measured using 405 nm excitation and detection from 410 to 470 nm. Three sequential imaging tracks were used, employing line‐switch scanning to alternate between channels efficiently. Pixel‐by‐pixel emission‐ratio analysis was conducted in batch mode using the specialised Redox Ratio Analysis software (Fricker, 2016).

### ER localisation analysis

Localisation of RTN17 puncta across the ER was manually identified and categorised into eight subdomains: within cisternae, within holes in cisternae, on cisternae edges, at cisternae‐tubule junctions, on tubules, at tubule edges, at three‐way junctions, and at the tip of elongating tubule.

### Actin and microtubule cytoskeleton co‐localisation analysis

The percentage of RTN17 co‐localising with the cytoskeleton was assessed by manually annotating RTN17 puncta relative to clearly resolved filamentous structures. Puncta were scored as “overlapping” when positioned directly on or at the edge of visible filaments, and as “non‐overlapping” when located in cytoplasmic regions lacking filament signal. Large aggregates of RTN17 puncta (area >359 nm^2^) were excluded from the analysis. Statistical significance was determined using an unpaired two‐tailed Student's *t*‐test.

### ER and cytoskeleton orientation analysis

High‐resolution confocal images were processed in Fiji (version 2.16.0/1.54p) (Schindelin et al., 2012) to reduce cytoplasmic background and enhance structure segmentation. For each channel, a Gaussian blur (radius 2.0) was applied, followed by contrast enhancement, median filtering (radius 2.0), and background subtraction. The effect of RTN17 on ER‐cytoskeleton alignment was analysed using CytoSpectre (Kartasalo et al., 2015), a MATLAB‐based software for measuring the orientation of fibre‐like structures in microscopy images. For each cell, the mean angular orientation of the detail component—a more accurate measure of the ER orientation network—of mTagBFP2‐HDEL, GFP‐TUA and GFP‐Lifeact was extracted, and the difference between the orientation of the ER and cytoskeletal structures was then calculated. Statistical comparisons were performed using two‐tailed Welch's *t*‐tests.

### AP‐FRET analysis

Acceptor photobleaching—Förster resonance energy transfer (AP‐FRET) was done in accordance with (Graumann, 2014). In short, regions of interest (ROIs) were imaged for 5 frames prior to bleaching with 100% 561 nm laser power via 20 iterations. The following 15 frames were then recorded. The first 5 frames had the GFP signal averaged to generate a pre‐bleach value and compared to the first post‐bleach value of the GFP fluorophore to generate the FRET efficiency as a percentage. 3 ROIs were measured per cell. RTN1‐eGFP and RTN1‐mRFP were used as a positive control due to previous confirmation via FRET‐FLIM (Kriechbaumer, Botchway, et al., 2015), and RTN17.1‐mRFP and GFP‐HDEL were used as a negative control.

### Analyser quantification of ER structure and dynamics (including constriction)

Quantification of the structure of the ER was performed according to (Pain et al., 2019). Briefly, confocal images and movies are loaded into the software. After an initial upsampling step, we selected the minimum and maximum widths of ER tubules (FWHM_max_ = 7, FWHM_min_ = 3) with a target width set between the two values. After initial filtering and background subtraction, cisternal regions are identified using an opening function. The tubular ER is enhanced using phase‐congruency analysis (Kovesi, 1999), and then each tubule is reduced to a single pixel wide skeleton running down the centre of each tubule. For movies, optical flow analysis using the Farnebäck algorithm is used to detect ER movement (Farnebäck, 2003).

Tubule constriction was analysed using an intensity trace along each tubule. Peaks and troughs in tubule intensity are identified using a peak finding algorithm applied to the intensity profile, or inverted intensity profile of tubules to detect peaks and troughs in intensity, respectively.

Results were analysed using the specialist analysis package that accompanies Analyser (Pain et al., 2019). After transformations of metrics to achieve normality as necessary, a parametric multivariate analysis (MANOVA) was used to identify whether the multivariate means came from different populations. Subsequently ANOVAs were applied comparing test groups against the control group mTagBFP2‐HDEL. To correct for multiple comparisons, a Bonferroni correction was applied to all *P*‐values produced by ANOVA.

### Rhodamine B staining and imaging

10‐day‐old Arabidopsis seedlings were submerged in 1 μm Rhodamine B hexyl ester for 15 min. They were then washed gently twice in dH_2_O and mounted in water prior to imaging. Samples were imaged using a 514 nm laser with emission detected at 470–500 nm.

## AUTHOR CONTRIBUTIONS

CM: Investigation, Data curation, Formal analysis, Validation, Writing. SW: Investigation, Data curation. VK: Funding acquisition, Conceptualization, Validation, Writing. CP: Validation, Conceptualization, Formal analysis, Funding acquisition, Writing.

## CONFLICT OF INTEREST

The authors declare no competing interests.

## Supporting information


**Figure S1.** The structure and localisation of RTN17 splice variants.
**Figure S2.** RTN17 hydropathicity and predicted TMDs.
**Table S1.** Comparison of the 405/488 nm intensity ratio of roGFP2 in different redox environments.
**Table S2.** RTN17 does not cause constriction of ER tubules on over‐expression.
**Table S3.** ER structural and dynamic changes that occur on transient over‐expression of RTN17, RHD3 and RTN17 and RHD3 together.
**Figure S3.** The effect of loss of expression of RTN17 in Arabidopsis.
**Figure S4.** ER structure analysis in Rhodamine B hexyl ester stained Arabidopsis cotyledons expressing RTN17‐Clover.
**Figure S5.** Optical flow analysis of ER dynamics.
**Figure S6.** ER dynamic changes after depolymerization of microtubules.
**Table S4.** Previously published constructs used in this work.

## Data Availability

The data that support the findings of this study are available from the corresponding author upon reasonable request.
