# Supplementary material for: An ER–microtubule bridge: Reticulon 17 links microtubules with ER network organisation in plants
Source: Plant J. 2026 Jul 4;127(1):e71028. doi: 10.1111/tpj.71028 (PMC13332829; doi:10.1111/tpj.71028)
Supplement: Supplementary file 1 — Figure S1. The structure and localisation of RTN17 splice variants. Figure S2. RTN17 hydropathicity and predicted TMDs. Table S1. Comparison of the 405/488 nm intensity ratio of roGFP2 in different redox environments. Table S2. RTN17 does not cause constriction of ER tubules on over‐expression. Table S3. ER structural and dynamic changes that occur on transient over‐expression of RTN17, RHD3 and RTN17 and RHD3 together. Figure S3. The effect of loss of expression of RTN17 in Arabidopsis. Figure S4. ER structure analysis in Rhodamine B hexyl ester stained Arabidopsis cotyledons expressing RTN17‐Clover. Figure S5. Optical flow analysis of ER dynamics. Figure S6. ER dynamic changes after depolymerization of microtubules. Table S4. Previously published constructs used in this work. [file TPJ-127-0-s001.docx]

**Supplementary Data**

**An ER–microtubule bridge: Reticulon 17 links microtubules with ER network organisation in plants.**

**Authors:** Carmen Mata, Stefan Wojcik, Verena Kriechbaumer, Charlotte Pain

**Supplementary Figure S1: The structure and localisation of RTN17 splice variants.**

Representative confocal images of over-expression of A) RTN17-Clover (green) transiently in tobacco leaf epidermal cells and stably in 10-day old Arabidopsis cotyledons. B) Schematic representations of the structure of the three naturally occurring RTN17 splice variants (RTN17.1, RTN17.2 and RTN17.3) with the common RHD highlighted in blue. Amino acid scale highlighted above the schematic. Transient over-expression in tobacco cells of the ER marker RFP-HDEL (magenta) and the RTN17 splice variants (green): RTN17.1-Clover C), RTN17.2-Clover D) and RTN17.3-Clover E). Scale bars = 5 μm.

**Supplementary Figure S2: RTN17 hydropathicity and predicted TMDs.**

A) A plot of the hydropathicity of RTN17 calculated using the method proposed by Kyte and Doolittle (Kyte and Doolittle 1982), with window size set to 12. B) Schematic representation of the TMDs (yellow) predicted by TOPCONS, OCTOPUS, Deep TMHMM in the RTN17 protein (blue) and their position relative to the RHD (red).

**Supplementary Table S1: Comparison of the 405nm/488nm intensity ratio of roGFP2 in different redox environments.**

Comparison of the 405nm/488nm intensity ratio of confocal images collected of two control constructs; roGFP2-HDEL which localises to the ER lumen and roGFP2 cytosol which localises to the cytosol. Also compared are the roGFP2 intensity ratios of N-terminally tagged RTN17 (roGFP2-RTN17) and C-terminally tagged RTN17 (RTN17-roGFP2). Also shown is the redox state of roGFP2 as predicted by the 405nm/488nm intensity ratio.

| Construct | Localisation | 405nm/488nm intensity ratio | Standard error (±) | Redox state |
| --- | --- | --- | --- | --- |
| roGFP2-HDEL | ER lumen | 1.23 | 0.03 | Oxidising |
| roGFP2 cytosol | Cytosol | 0.39 | 0.02 | Reducing |
| roGFP2-RTN17 |  | 0.55 | 0.04 | Reducing |
| RTN17-roGFP2 |  | 0.39 | 0.02 | Reducing |

**Supplementary Table S2: RTN17 does not cause constriction of ER tubules on over-expression.**

Key constriction analysis metrics comparing peaks and troughs in intensity of CXN-mCherry, RTN1-mRFP and RTN17-mRFP along ER tubules in comparison to changes in the lumenal marker GFP-HDEL intensity. The p-values of an initial ANOVA comparison across each group are shown, alongside mean results for each measurement and the standard errors of each measurement. Data collected from n=3 biological replicas and n=25, CXN-mCherry; n=26, RTN1-RFP and n=30, RTN17-RFP technical replicas.

|  | ANOVA p-value | CXN-mCherry  (mean ± SE) | RTN1-mRFP  (mean ± SE) | RTN17-mRFP  (mean ± SE) |
| --- | --- | --- | --- | --- |
| Peak ratio | 2 x 10^-16^ | 1.28 ± 0.058 | 13.6 ± 1.45 | 0.720 ± 0.04 |
| Trough ratio | 1.05 x 10^-11^ | 1.03 ± 0.046 | 13.0 ± 2.16 | 0.132 ± 0.02 |

**Supplementary Table S3: ER structural and dynamic changes that occur on transient over-expression of RTN17, RHD3 and RTN17 and RHD3 together**.

Comparison of key ER structural and dynamic metrics, including tubule length, cisternal area and geometry, polygonal region area and geometry and tubule and cisternal speeds. The F-statistic and p-value of a planned comparison to each condition and the control condition are shown. p-values are adjusted for multiple comparisons with a Bonferroni correction. Non-significant results are shown by NS, whilst significant results are denoted by p-value 0.05-0.001 = *, p-value 0.001-0.001 = ** and p-value ≤ 0.001 = ***. The results of post hoc analysis (Tukey’s HSD) are included for any analysis where there is a significant ANOVA result.

| ER subdomain | Parameter | ANOVA | | | Post hoc analysis | | |
| --- | --- | --- | --- | --- | --- | --- | --- |
|  |  | F | Adjusted p-value | Significance | Control vs RHD3- Clover | Control vs RTN17-mRFP | Control vs RHD3-Clover + RTN17-mRFP |
| Tubule | Length | 1.26 | 0.3 | NS |  |  |  |
|  | Area | 2.41 | 0.076 | NS |  |  |  |
|  | Tortuosity | 2.8 | 0.048 | * | 0.9694 | 0.948 | 0.0204 |
|  | Maximum speed | 8.99 | 6.13x10^-5^ | *** | 0.1119 | 0.0425 | 0.2168 |
|  | Mean speed | 7.08 | 4.17x10^-4^ | *** | 0.1081 | 0.1635 | 0.3573 |
|  | Mean persistency | 3.48 | 0.022 | * | 0.0262 | 0.9824 | 0.9892 |
| Cisternae | Contrast | 0.91 | 0.44 | NS |  |  |  |
|  | Correlation | 7.28 | 3.33x10^-4^ | *** | 0.9992 | 7.42x10^-4^ | 0.4112 |
|  | Energy | 2.88 | 0.044 | * | 0.9876 | 0.0564 | 0.9882 |
|  | Homogeneity | 1.01 | 0.4 | NS |  |  |  |
|  | Circularity | 0.2 | 0.89 | NS |  |  |  |
|  | Elongation | 0.38 | 0.77 | NS |  |  |  |
|  | Roughness | 0.12 | 0.95 | NS |  |  |  |
|  | Area | 0.77 | 0.51 | NS |  |  |  |
|  | Major axis length | 0.41 | 0.75 | NS |  |  |  |
|  | Minor axis length | 1.3 | 0.28 | NS |  |  |  |
|  | Perimeter | 1.32 | 0.28 | NS |  |  |  |
|  | Solidity | 0.25 | 0.86 | NS |  |  |  |
|  | Maximum speed | 7.85 | 1.88x10^-4^ | *** | 0.139 | 0.0461 | 0.2496 |
|  | Mean speed | 9.79 | 2.84x10^-5^ | *** | 0.0481 | 0.0541 | 0.1637 |
|  | Mean persistency | 6.06 | 1.23x10^-3^ | ** | 0.8032 | 0.2657 | 0.02 |
| Polygonal regions | Area | 1.27 | 0.3 | NS |  |  |  |
|  | Eccentricity | 20.04 | 6.00x10^-9^ | *** | 0.606 | 0.5401 | 9.84x10^-6^ |
|  | Major axis length | 0.47 | 0.7 | NS |  |  |  |
|  | Minor axis length | 2.39 | 0.078 | NS |  |  |  |
|  | Perimeter | 0.8 | 0.5 | NS |  |  |  |
|  | Solidity | 4.62 | 0.0059 | ** | 0.5661 | 0.004 | 0.8056 |
|  | Convex area | 1.19 | 0.32 | NS |  |  |  |
|  | Circularity | 1.39 | 0.26 | NS |  |  |  |
|  | Roughness | 3.52 | 0.021 | * | 0.9884 | 0.3853 | 0.375 |
|  | Elongation | 15.94 | 1.30x10^-7^ | *** | 0.8259 | 0.9857 | 1.70x10^-5^ |

**Supplementary Figure S3: The effect of loss of expression of RTN17 in Arabidopsis.**

Representative images 10-day old Arabidopsis cotyledons stained with Rhodamine B to visualise the ER from stable Arabidopsis lines A) Col-0 and B) T-DNA insertion line, *rtn17.* Yellow boxes indicate the close-up sections (Ai, Bi, Bii), with tubule nodules indicated by red arrows. C) Comparison of nodules (0.3-0.4 μm^2^) per cell area and D) tubule lengths in Arabidopsis lines lacking RTN17 expression. * Denotes significant results (post hoc Tukey test, p-value = 0.039) and ns, non-significant results. Results taken from Col-0, n=27 and *rtn17*, n=22 from 3 biological replicas. Scale bars = 5 μm.

f)

| ER subdomain | Parameter | ANOVA | | |
| --- | --- | --- | --- | --- |
|  |  | F | Adjusted p-value | Significance |
| Tubules | Length | 2.15 | 0.15 | NS |
|  | Tortuosity | 1.78 | 0.19 | NS |
| Cisternae | Area | 3.68 | 0.061 | NS |
| Polygons | Area | 0.021 | 0.89 | NS |
|  | Elongation | 0.52 | 0.48 | NS |

**Supplementary Figure S4: ER structure analysis in Rhodamine B hexyl ester stained Arabidopsis cotyledons expressing RTN17-Clover.**

A) Representative image of Rhodamine B hexyl ester stained RTN17-Clover cell of 10-day old Arabidopsis cotyledons after filtering. B) Identification of the skeletonised tubular network (magenta) and identified cisternae (yellow) by AnalyzER. C) Example quantification of the tubule length shown as a graphical representation overlayed on the original image. D) Calculated tubule widths pseudo-coloured and overlayed on an inverted original image. E) Identified polygonal regions that are fully enclosed by the network (magenta) with cisternae outlined in black. Scale bars = 5µm.

F) Data comparison of example ER morphological metrics from AnalyzER: tubule length and tortuosity, cisternal area, polygonal region area and elongation. The F-statistic and p-value of an ANOVA are shown. p-values are adjusted for multiple comparisons with a Bonferroni correction. Non-significant results are shown by NS. Results taken from Col-0, n=27 and RTN17-Clover, n=21 from 3 biological replicas.

**Supplementary Figure S5: Optical flow analysis of ER dynamics.**

Representative pseudo-colour coded images of ER dynamics in tobacco epidermal cells. The ER is visualised using A) mTagBFP2-HDEL alongside B) RHD3-Clover, C) RTN17-mRFP, D) both RTN17-mRFP and RHD3-Clover. Images are pseudo-coloured based on the included scale ranging between 0μm s^-1^ (blue) and 2.5 0μm s^-1^ (red). Scale bars = 5 μm.

| ER subdomain | Parameter | ANOVA (RTN17 × oryzalin) | | |
| --- | --- | --- | --- | --- |
|  |  |  |  |  |
|  |  | F | Adjusted p-value | Significance |
| Tubule | Maximum speed | 2.81 | 0.1 | NS |
|  | Mean speed | 4.05 | 0.05 | * |
|  | Mean persistency | 10.64 | 0.002 | ** |
| Cisternae | Maximum speed | 4.73 | 0.035 | * |
|  | Mean speed | 1.52 | 0.22 | NS |
|  | Mean persistency | 3.96 | 0.52 | NS |

C)

D)

| Treatment | Construct | Mean parameter | |
| --- | --- | --- | --- |
|  |  | Mean tubule speed | Maximum cisternal speed |
| DMSO | TUA | 0.11 ± 0.01 | 0.39 ± 0.06 |
|  | RTN17 + TUA | 0.11 ± 0.02 | 0.39 ± 0.07 |
| Oryzalin | TUA | 0.15 ± 0.02 | 0.45 ± 0.06 |
|  | RTN17 + TUA | 0.13 ± 0.03 | 0.41 ± 0.06 |

**Supplementary Figure S6: ER dynamic changes after depolymerization of microtubules.**

A–B) Representative images of tobacco leaf epidermal cells 45 min after treatment with 10 µM oryzalin to induce microtubule depolymerization. Cells transiently express mTagBFP2-HDEL (white), GFP-TUA (green), and RTN17-mRFP (magenta) alongside the merged image. Depolymerisation of microtubules disrupts the linear distribution of RTN17 puncta observed under control conditions. A) Cell showing complete microtubule depolymerisation. B) Cell showing partial microtubule depolymerisation, with residual microtubule structures still visible. C) Means of results of the two-way ANOVAs testing the effects of RTN17 and oryzalin treatment interaction (RTN17 × oryzalin) on ER dynamic parameters. F-statistics and Bonferroni-adjusted p-values are shown. D) Mean tubule speed and maximum cisternal speed of groups. Data were collected from at least n = 3 biological replicates and n = 10 technical replicates. Non-significant results are shown by NS, whilst significant results are denoted by p-value 0.05-0.001 = * and p-value 0.001-0.001 = **.

**Supplementary Table S4: Previously published constructs used in this work.**

A list of previously published plant expression plasmids used as part of this work, along with relevant references.

| **Construct** | **Reference** |
| --- | --- |
| GFP-HDEL | (Brandizzi et al., 2003) |
| roGFP2-HDEL | (Schwarzländer et al., 2008) |
| roGFP2 cytosolic | (Wang et al., 2011) |
| CXN-mCherry | (Groves *et al.*, 2019) |
| RTN1-mRFP | (Sparkes *et al.*, 2010) |
| GFP-Lifeact | (Riedl et al., 2008) |
| GFP-TUA | (Ueda et al., 1999) |
| mTagBFP2-HDEL | (Li et al., 2022) |
| mRFP-HDEL | (Brandizzi *et al.*, 2003) |
